# Acceptability of Carraguard, a candidate microbicide and methyl cellulose placebo vaginal gels among HIV-positive women and men in Durban, South Africa

**DOI:** 10.1186/1742-6405-4-20

**Published:** 2007-09-27

**Authors:** Gita Ramjee, Neetha S Morar, Sarah Braunstein, Barbara Friedland, Heidi Jones, Janneke van de Wijgert

**Affiliations:** 1 Medical Research Council, P O Box 70380, Overport, 4067, Kwazulu-Natal, South Africa; 2 Population Council, One Dag Hammarskjold Plaza, New York, New York, 10017, USA; 3 Academic Medical Center, Center for Poverty-related Communicable Diseases, University of Amsterdam, Meibergdreef 9, PO Box 22700 1100 DE Amsterdam, the Netherlands

## Abstract

**Background and Methods:**

When on the market, microbicides are likely to be used by individuals who do not know their HIV status. Hence, assessment of safety and acceptability among HIV positive men and women is important. Acceptability of Carraguard, the Population Council's lead microbicide candidate was assessed in a Phase I safety study among healthy HIV-positive sexually abstinent women and men, and sexually active women (20 per group), in Durban, South Africa. Participants were randomized to use Carraguard gel, placebo gel, or no product. All women in the gel arms applied 4 ml gel vaginally every evening for 14 intermenstrual days (women in the sexually active group inserted gel within 1 hour prior to sex on days when sex occurred), and sexually abstinent men applied gel directly to the penis every evening for 7 days. Acceptability was assessed by face-to-face structured questionnaires and semi-structured in-depth interviews with all participants. Gel use questions were applicable to participants in the gel arms only (13 sexually abstinent women, 14 sexually active women, and 13 abstinent men).

**Results:**

Overall, 93% of the women liked the study gel (Carraguard or placebo) very much, 4% disliked it somewhat, and 4% were neutral. 15% of men and women disliked the gel's color, smell, or packaging. Most women and men reported never experiencing pain or irritation during or after gel application. Although over two thirds of the women preferred some lubrication during sex, some of the women felt that the gel was frequently too wet. Twenty-one percent of women and 42% of men said they felt covert use of a microbicide would be acceptable. Over 60% of women and men would prefer to use a microbicide alone instead of using it with a condom.

**Conclusion:**

Acceptability of Carraguard among HIV-positive women and men in Durban was good. The wetness experienced by the women may be attributed to the delivery of gel volume. The applicator was designed to deliver 4 mls whereas in fact between 4 ml to 5 mls were actually dispensed. Condom migration in the event of a partially effective product is of concern.

## Background

Vaginal microbicides are products designed to provide women, particularly those who are unable or unwilling to use male condoms, with the means to protect themselves against sexually transmitted infections (STI), including HIV infection. Since the 1990s, research and development of microbicides has grown in response to the alarming rate at which women and girls around the world are becoming infected with HIV and AIDS, particularly in resource-limited settings [1,2]. While this new class of products has the potential to make an important contribution to efforts to curb the spread of HIV/AIDS, the ultimate effectiveness of microbicides will hinge on whether and how often individuals who are at risk for HIV infection use them. For this reason, the development of products that are acceptable to users is critical for ensuring that microbicides achieve their public health objective of preventing new cases of HIV infection.

There is growing consensus in the microbicides field that the safety and acceptability of promising candidate products should also be evaluated in HIV-infected women and men [3]. We believe that once on the market, women and couples may use a microbicide without knowing their HIV status. Furthermore, HIV positive women and couples may want to use a microbicide to prevent infection with other strains of HIV, to prevent acquisition of other sexually transmitted infections (STI), or to protect their sexual partner(s) from getting infected. Furthermore, it is important for this population to know that this product could be used by them.

Many microbicide studies are undertaken to assess safety and acceptability among sexually active and abstinent women. However there is paucity in the data on safety and acceptability of the potential microbicides among HIV positive women and men. The present study was undertaken to assess the safety and acceptability of Carraguard among HIV positive sexually active and abstinent women and sexually abstinent men. The outcome of the safety paper is published separately [4] whereas the focus of this paper is on the acceptability of the microbicide in this population. Given that a phase III trial of Carraguard has just been completed, it is important to assess acceptability of the product in diverse population groups to obtain some information on safety and acceptability.

### Methodology

A Phase I randomized, controlled trial was conducted from May 2002 to July 2003 to evaluate the safety and acceptability of Carraguard among HIV positive sexually abstinent men and sexually active and abstinent HIV-positive women in Durban, South Africa. The study was designed to assess safety and acceptability among HIV positive sexually abstinent men and women first to minimize any risk of transmission to sexual partners. This was followed by assessment of safety and acceptability among sexually active women. The detailed study methodology has been published elsewhere [4].

Briefly, HIV-infected women and men were recruited from clinics, hospitals, and support groups in the Durban area that provide HIV care and treatment. Women were eligible if they were aged 18 to 45 years, and were HIV positive but otherwise in good health as determined by medical history and physical examination (including CD4+ cell count > 200 × 10^6^/L and absence of opportunistic infections, vaginal infections, STI, or genital abnormalities). Women enrolled into the sexually active study group had to be sexually active (defined as sexual intercourse at least twice per week) and willing to have sex with only one male partner who was also HIV positive, during the study. Men were eligible to participate if they were aged 18 or older, were confirmed HIV positive, and were in good health otherwise (same criteria as women) and were willing to stay sexually abstinent for the duration of their participation. Participants were asked to visit the clinic at screening and after enrollment women returned to the study clinic for evaluation at Days 7, 14, and 21 (7 days after cessation of gel use). After enrolment male participants were seen at day 7 and day 14 (7 days after cessation of gel use). Procedures followed at each visit are described in detail elsewhere [4]. All participants provided written informed consent for study participation at screening and enrollment. The study was approved by local and Population Council ethics review committees.

**Figure 1:**
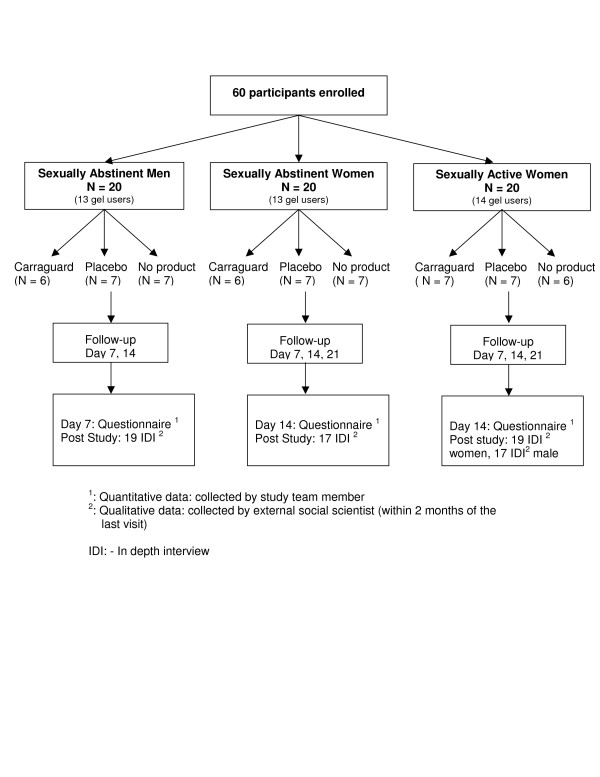
Randomization scheme and study assessments.

Sixty participants were enrolled into three study groups (Figure 1). 20 participants were enrolled in the sexually abstinent women group; 20 in the sexually active women group; and 20 in the sexually abstinent men group. Within each study group, participants were randomized to one of three study arms: Carraguard, methylcellulose placebo, or no product (Figure 1). Condoms and safe sex counselling were provided for participants in all groups at all study visits.

The sexually inactive cohort was selected because we wanted to assess the safety in sexually abstinent men and women first and then in sexually active couples. This would allow us to ascertain the safety of the product in the absence of sexual activity which may in itself cause some epithelial disruption. Although the product is now in Phase III trial, it was important to assess if there were any safety concerns among HIV positive individuals. The no product arm was in the design of the study because the placebo gel may also cause certain safety outcomes such as genital findings, thus it was necessary to ascertain safety in the absence of any product.

At the enrollment visit for women and men, and again at Day 7 for women, participants who were randomized to a gel-use group were given a box of 8 single-dose Microlax^®^-type applicators containing either Carraguard or placebo (2.5% methylcellulose). Applicators contained 7 ml of gel each, and were designed to dispense approximately 4 ml at a single use, however 4–5 ml was dispensed and adherence and acceptability was measured on this volume. All women were instructed to insert the gel into the vagina each evening for 14 days; women in the sexually active group were instructed to use the gel up to 1 hour prior to sexual intercourse or in the evening on days when sex did not occur. Men were instructed to apply the gel directly to the penis each evening for 7 days, and to leave the gel on overnight. We considered 7 days of exposure to be sufficient as men are only exposed briefly to the gel during sex and could wipe off the gel immediately after sex unlike women. Adherence to gel use was measured on the men and women's compliance to the given instructions

Acceptability of Carraguard and the placebo gel was assessed both quantitatively and qualitatively for study participants (Figure 1). Quantitative data on male partners of sexually active women was not collected. Quantitative acceptability data were collected via face-to-face structured interviews conducted in the local isiZulu language by a study team member (Figure 1). Questionnaires were administered to all participants at the end of the gel-use period (Day 14 visit for women and Day 7 visit for men). The questionnaire contained approximately 70 questions that covered a range of parameters such as participants' opinions of product attributes, their experience of product use, sexual behaviour and preferences, and attitudes toward condoms and vaginal microbicides (Table 1). Furthermore, all participants and the consenting male partners of women in the sexually active study group were invited to participate in a semi-structured in-depth interview (IDI) with an external social scientist within two months of their final study visit. All invited males from each of the study arms were invited to ascertain their views of microbicides. Of note is that both hypothetical acceptability (for partners of women on no product arm) and real use acceptability was assessed. All male partners were consented, and offered voluntary counseling and testing for HIV. A total of 72 IDI were conducted among: 19 of 20 sexually abstinent men, 17 of 20 sexually abstinent women, 19 of 20 sexually active women, and 17 of 20 male partners of sexually active women (Figure 1). We ensured that an external social scientist conducted the interviews to minimize social desirability. However, this was only done with male partners of sexually active women.

**Table 1:** Acceptability Questionnaire: Parameters and sample questions

**Parameter**	**Number of questions**	**Sample question**
*Gel use experience and product attributes*	26	Did the gel dry out too quickly?
		Was the gel too wet or drippy?
		How did you feel about the amount of gel you had to insert/use each time?
		How did you feel about inserting the applicator into your vagina?
		How would you rate the study product (gel and applicator) overall?
*Covert use of microbicides*	2	Do you think women, in general, could use this gel during sex without their partners' knowledge?
*Condom and gel use during sex in study*	9	If there were times during the study that you used condoms, what was the main reason?
		On average, how long before sex did you insert the gel?
*Sexual preferences*	2	Do you prefer sex to be: very dry, dry, somewhat lubricated, very lubricated, or no preference?
*Attitudes toward potential microbicides*	26	What kind of microbicides do you think women in your community need?
		Would you be more willing to use a microbicide if it protected against both HIV/STDs and pregnancy, or only HIV/STDs?
		How important is a product that makes your vagina feel clean?

### Statistical analysis

IDI data were coded by two staff members at the Population Council in New York, and analyzed using the ATLAS.ti^® ^software package for qualitative data (ATLAS.ti GmbH Company, Berlin, Germany). Codes were based on product characteristics, themes such as influence of gel use on sexual behaviour, and preferences for HIV/STI prevention methods, including vaginal microbicides. Data were coded using thematic analysis to assess patterns and variability in participants' responses to interview questions.

Quantitative data from the questionnaires were double-entered into an Access database (Microsoft, Inc., Redmond, Washington, USA) and analyzed using the SPSS statistical software package (SPSS, Inc., Chicago, Illinois, USA). All participants completed the acceptability questionnaire.

## Results

Our findings showed that there was no difference in the response between reported acceptability of Carraguard compared to placebo groups hence combined results of gel acceptability are thus presented.

### Baseline demographics and sexual behavior

The mean age of all enrolled women and men was 29 years (range 19–43) and 32 years (range 21–50) respectively (Table 2). Women had a mean of 12 years of education compared to 10 years for men. The majority of women and men were legally married or in the process of getting married (defined as some *lobola *or "bride price" having been paid), or were in a steady partnership. Ninety percent of enrolled women reported having given birth in their lifetime.

**Table 2:** Baseline demographics and sexual behavior

	**Sexually Abstinent women (n = 20)**	**Sexually active women (n = 20)**	**Sexually abstinent men (n = 20)**
Median age in years (range)	29.5 (19–42)	28 (22–41)	31.5 (21–50)
Mean years of education (range)	12 (6–15)	11.5 (5–14)	10 (4–16)
Married/steady partner	80% (16/20)	100%	90% (18/20)
Ever gave birth	90% (18/20)	90% (18/20)	*Not applicable*
Children with one partner	60% (12/20)	70% (14/20)	*Not applicable*
Median number live births (range)	1.50 (0–5)	1.50 (0–5)	*Not applicable*
Vaginal sex in last month (proportion)	55% (11/20)	100% (20/20)	65% (13/20)
Mean number vaginal sex acts in last month (range)	2.7 (1–4)	5.5 (1–18)	5.2 (1–30)
Male condom use in last month (proportion)	64% (7/11)	90% (18/20)	62% (8/13)
Mean number lifetime sex partners (range)	3.1 (1–15)	3.2 (1–6)	15.2 (3–90)
Ever tested for HIV (proportion)	85% (17/20)	100% (20/20)	80% (16/20)

The majority of women (78%) reported having had vaginal sex in the month prior to enrollment, with an average number of 2.7 sex acts in sexually abstinent woman and 5.5 in sexually active women (Table 2). Most women (81%) reported having used a condom at least once during vaginal sex in the month prior to enrollment. Among men, nearly two thirds (65%) reported having had vaginal sex in the month prior to enrollment, with an average number of sex acts of 5.2 (Table 2). More than half of the men (62%) reported having used a condom at least once during vaginal sex in the previous month. Almost all women (93%) and men (80%) had been tested for HIV prior to study participation and were aware of their HIV status at study enrollment.

### Overall product rating

In general, participants expressed favorable opinions about the study product (herein defined as study gel plus applicator). More than 90% of female and male gel users reported that they liked the study product overall, and most of these participants reported that they liked the gel "very much" (Table 3). Only one sexually active woman gel user reported disliking the product somewhat as she felt the gel was cold, preferred the color to be pink and suggested that the gel be inserted twice a week only rather than at every sex act. In the context of such favorable ratings of the study product, adherence to product use among women was 100% and all but one man complied with product use.

**Table 3:** Participants' attitudes toward study products

	**All women (n = 27)**	**Men (n = 13)**
**Overall study product rating:**		
• Liked	93% (25/27)	92% (12/13)
• Felt neutral	4% (1/27)	8% (1/13)
• Disliked	4% (1/27)	0
Liked gel's color	89% (24/27)	92% (12/13)
Liked gel's smell	85% (23/27)	69% (9/13)
Liked amount of gel	74% (20/27)	54% (7/13)
Liked gel's packaging	89% (24/27)	*Not applicable*
Liked carrying applicators with you	67% (18/27)	46% (6/13)
Gel ever^1 ^too wet	67% (18/27)	54% (7/13)
Gel ever too sticky	26% (7/27)	39% (5/13)
Gel ever come out of applicator too quickly	44% (12/27)	62% (8/13)
Gel ever soiled woman's clothes^2^	63% (17/27)	*Not applicable*
Prefers lubrication during sex^3^	82% (32/39)	*Not applicable*
Gel ever caused pain or irritation while using it	7% (2/27)	0
Gel ever caused pain or irritation after using it	4% (1/27)	0
Women in general could use gel without partner's knowledge	30% (8/27)	54% (7/13)
Woman herself would use gel without partner's knowledge	26% (7/27)	*Not applicable*
Would buy gel to use with spouse/steady partner	100% (27/27)	100% (13/13)

1. Here, "Ever" includes the responses rarely, sometimes, often, almost always, and always.

2. Note: we also asked participants about the gels drying too quickly and being too sticky, and whether the gels came out of the applicator too slowly. Very few users reported any of these as a problem.

3. All women (N = 39), regardless of randomisation group, were asked this question.

### Product characteristics

Participants were asked for their opinions on a range of gel and applicator characteristics, including the color, smell, volume and consistency of the gels, and the design and function of the gel applicator. The following sections describe participants' responses to particular features of the study products.

#### Gel characteristics: Smell/color

In general, participants liked the gel's neutral color and smell. Eighty-nine percent (24/27) of the women and 92% (12/13) of the men reported that they liked the clear color of the gels (Table 3). In addition, 85% (23/27) of the women and 69% (9/13) of the men reported that they liked that the gels were odorless.

Hypothetical acceptability among participants who were not randomized to a gel-use group expressed a preference for a product with a neutral color and smell. For example, one sexually active woman in the no product arm said: *"I think that what should be inserted in the vagina must not have [a] smell [or] color."*

#### Gel characteristics: Applicator/packaging

Eight-nine percent (24/27) of the women gel users approved of the products' packaging and applicator.

#### Gel characteristics: Volume/consistency

Over two thirds of the women gel users liked the amount of gel the applicator dispensed, with 54% (7/13) of men in agreement (Table 3). Very few participants felt the gel was ever too sticky, or that the gel dried too quickly after application. Many women felt that the gel dispensed very quickly from the applicator. However, a third of the women participants felt the gel was too wet during or after use. This was probably due to larger volume actually dispensed trhough the applicator then expected.

### Pain/irritation during or after product use

Very few participants reported experiencing side effects from the gels, such as pain or irritation during or after gel use. Only 2 women (7%) reported any pain or irritation during gel use, and 1 woman (4%) reported pain or irritation after use; none of the male users experienced side effects. A woman randomised to Carraguard reported, *"I did not see anything wrong... I was expecting that I might get side effects in the vagina but I did not."*

### Effects of gel use on sex

Sexually active participants were asked about their experience of using the gel during sex. In general, their responses were favorable. All sexually active women gel users reported they always remembered to use the gel before sex. Seventy-one percent (10/14) of these participants reported that the gel made sex more pleasurable and less painful, with 36% (5/14) reporting that the gel increased their frequency of sexual intercourse. Their male partners also had positive feelings about the gel, and many of them reported a preference for the gel over condoms. Finally, there was a strong desire for the ideal microbicide to enhance sexual pleasure for both partners (Table 4).

**Table 4:** Traits of an ideal microbicide

**Important^1 ^for microbicide to:**	**All women (N = 39)**	**Men (N = 19)**
Add extra lubrication during sex	69% (27/39)	58% (11/19)
Dry the vagina	41% (16/39)	37% (7/19)
Make the vagina feel warm	90% (35/39)	79% (15/19)
Make the vagina feel tight	67% (26/39)	74% (14/19)
Make your/your partner's vagina feel clean	95% (37/39)	95% (18/19)
Increase your own sexual pleasure	100% (39/39)	84% (16/19)
Increase your partner's sexual pleasure	90% (35/39)	84% (16/19)
Not be noticed by you	90% (35/39)	90% (17/19)
Not be noticed by partner	85% (33/39)	84% (16/19)
Smell good	77% (30/39)	68% (13/19)
Taste good	51% (20/39)	47% (9/19)

1. Here, "Important" combines the responses very important and somewhat important.

#### Timing of gel use and adherence

We did not measure the median timing of gel use in relation to sex but asked the women if they were able to apply the gel within an hour prior to sex. However, reported adherence with gel use was high [4].

Overall, sexually active women reported inserting the gel on average 61 minutes before sex. Women seemed to accept that the product was to be used at the time of intercourse. For example, one sexually active woman in the placebo group said: "*I think it is okay that you insert it when you are going to have sex." *Although women were able in general to adhere to the timing of gel use, they were not always able to adhere to gel use as per instruction. This may reflect the difficulty of controlling the timing of sex for some women. For example, one sexually active woman in the Carraguard group said: *" [the] instruction was to apply [the gel] one hour before having sex but it [was] hard to control it because you don't [know] when can you be ready for sex."*

Male partners were also asked about the timing of gel use in relation to intercourse during the IDI. Many of the partners seemed to accept the timing of gel use. For example, one partner of a woman in the placebo group said: *"I do not see time and the manner in which it is applied to be [a] problem." *Other male partners expressed more complex opinions about the timing of product use. A partner of another woman in the placebo group said: *"it is right if [the gel] settles first... I think [my partner] needs to take sometime after she has inserted [the gel]. [It] will be better if it can be like... when she is going to have sex in the evening [the woman can] insert [the gel] in the afternoon maybe three hours before... It must be something that is inserted when we want to have sex."*

#### Effect on lubrication during sex

The preference for dry sex that has been reported by participants in other research studies in sub-Saharan Africa [5] was not commonly expressed by these participants. For example, only 18% (7/39) of women expressed a preference for sex in general to be dry. Further, many participants felt the ideal microbicide should add some lubrication during sex, and fewer felt it should dry the vagina (Table 4). Most women in the sexually active group (93% or 13/14) felt that the gels did add lubrication during sex, and most (70% or 10/14) felt this was an advantage, with a number of participants reporting that the added lubrication made sex more enjoyable and even less painful for some. Many of their male partners also felt that the added lubrication during sex from the gel was an advantage.

" [The] gel is slippery and it's alright... It makes sex enjoyable." [Sexually active woman, Carraguard]

### Covert use and other product use dynamics

The majority of the respondents felt that the product was easy to use. All of the sexually active women gel users and 11 out of 13 of the sexually abstinent women gel users said they liked inserting the applicator into their vagina. Similarly, 10 out of 13 of the sexually abstinent men gel users (77%) said they liked applying the gel to their penis. These favorable opinions regarding the ease of gel use were also expressed during the IDI:

" [The applicator] was all right and it was the right size. It was neither small nor big... You open it and [the gel] becomes squeezed out easily." [Sexually abstinent woman]

A few respondents did mention during the IDI that they had difficulty finding a private space for gel use, as reflected in this participant's comment:

"We got used to [inserting the gel] even while it was difficult because you have to be alone and avoid many people. [Finding privacy is] something that is not easy to do." [Sexually abstinent woman]

#### Covert use of a microbicide

In general, relatively few respondents felt that a microbicide could – or should – be used covertly. Twenty-one percent (3/14) of the sexually active women gel users felt that the gel could be used without women's partners knowing, but only 14% (2/14) reported that they would use the gel covertly. Support for covert use was stronger among sexually abstinent women, with 39% (5/13) saying that women in general could use the gel without their partner knowing, and 39% reporting that they would use the gel covertly. Interestingly, somewhat more men than women were supportive of covert use of a microbicide, with 42% (8/19) saying that women in general should be able to use the gel covertly, and 54% (7/13) saying they felt the gel could be used by women without their partner knowing. The majority of women (74%) and men (79%) felt that the decision to use a microbicide should be made by a woman and man together.

### The need for microbicides

Many of the respondents expressed a strong need for microbicides as another option for protection from HIV and potentially other STI. All of the women in the gel-use groups reported that they would purchase the gel to use with their husband or steady partner, and all of the men said they would buy the gel for their wife or steady partner to use. All male and female participants also said they would recommend the gel to a friend. Some of their comments during the IDI on this topic included the following:

"My opinion about [using the gel] is [it would be favorable if] you will be able to talk with your partner knowing each other's HIV status... [and] there is something that can be a protection measure that will serve as if nothing has happened [to] the married couple." [Male partner who also participated in sexually abstinent cohort]

"I wanted to be part of [this research] so as to help the community [and] and the people for instance, maybe there is a gel or something that might arise that will help the people who have HIV..." [Sexually abstinent man]

"It will help in that HIV infected persons won't infect others." [Sexually active woman, Carraguard]

Participants also discussed the importance of having an alternative to condoms. For example, one sexually active woman in the placebo arm said: *" [M]ost men do not want to use condoms and the man I am with right now I am not sure that I'll be with him for the rest of my life. It may happen that [I] separate with him and I meet someone who doesn't want condoms and we realize that we love each other, so we can use the gel if he doesn't want to use condoms. So that's what I liked about the gel because it can help you if you don't use a condom."*

A woman in the no product arm commented: *"I will be happy if [the] gel is successful. It will be better than condoms because condoms burst at times."*

## Discussion

In high HIV prevalence and incidence areas where microbicides are likely to be introduced for HIV Prevention, it is imperative that acceptability of novel technologies is determined in the population at large. Our study suggests the need and acceptability of microbicides among those who are already infected with HIV. The strengths of the study include: a) that Carraguard is in advanced stages of clinical testing and so acceptability data are particularly important; b) it is one of the few studies to assess acceptability in a randomised controlled trial in sexually active and abstinent HIV positive women, and sexually abstinent HIV positive men; and c) it adds value to current knowledge on microbicide acceptability by assessing acceptability and perceptions based on use of product in advanced Phase III trials.

From the responses received by both HIV-positive men and women in the current study regarding the need for microbicides, it is suggested that irrespective of individual HIV status, a product such as a microbicide would be purchased, used and accepted by individuals. This study confirms favorable acceptability findings from previous studies among men and women [6,7].

One of the disconcerting findings of this study and other studies involving men is that we are likely to see condom migration in the event a microbicide or any other intervention becoming available. Given that many of these products are unlikely to be 100 % effective, accurate messages regarding the use of the product with condoms will need to be emphasized. Behaviour change including promotion of safe sex using condoms will have to be emphasized.

Adherence to product use and its coital dependency is an important issue in ongoing large scale trials. In this short study, adherence to product use was very good among all study participants. The study was too short to ascertain whether this trend would have continued for long-term use. Of note, however, was that application of the product within the time-frame of 1 hour prior to sexual intercourse may not always be achievable and it would be important to develop products which are longer acting. While these findings suggest that coital dependency of the product may not be an issue if the product is easy to use and acceptable, it is important to bear in mind that some women are not able to control the timing or terms of sex and so may have difficulty using a product that requires use just prior to intercourse. From these data, it appears that product use would be enhanced if a woman's partner is aware and accepting of the product as well.

Unlike other studies in sub-Saharan Africa that have reported a common preference for "dry sex" [8], it appears that our study population did not have a high desire for dry sex and that some lubrication provided by the gel was acceptable. However, it was clear, too, that a significant number of participants felt the gel caused excessive wetness, and so the volume of gel may have been too much. While the applicators were designed to deliver 4 ml of gel, data from earlier studies using the same applicator indicated that 4.5–5.0 ml was the average amount squeezed out, which may have been why women found the gel to be too wet [7,9] Despite this concern, participants did report favorably on the effect of the gel on sex. This is in line with previous studies that showed that vaginal gel can enhance sexual pleasure [10,11].

When the need for microbicide products discussions began in the late 1980's [12] it was strongly believed that a product that a woman could use covertly was required to prevent HIV acquisition among women and that women's empowerment was key to addressing the escalating HIV infection among women. However, over the years, it has become increasingly evident in hypothetical and real-use acceptability studies among both men and women that covert use may not be desirable for all women, particularly those in steady relationships. In this study, less than a third of the women felt that the gel could be used without a male partner's knowledge, and only 26% of them said they themselves would use the gel covertly. Interestingly, somewhat more men (around half) said they thought women should or could use the gel covertly. However, the majority of the men did respond that use of the product should be a joint decision, which is in keeping with other research from South Africa. It is difficult to pinpoint the precise dynamics underlying these mixed responses. It is possible that the low preference for covert use among these individuals reflects the types of relationships (e.g., steady vs. casual, whether egalitarian, etc.) they are in. Alternatively, it could be linked to participants' other opinions about the gel, for example the feeling that the gel was too wet, which would logically reduce confidence in being able to use such a product covertly. Participants' strong desire for an ideal microbicide "not to be noticed" could also be related to their opinions about covert use (Table 4). Despite these findings, however, the possibility to use a microbicide covertly remains an important option for those who would need it.

While it is desirable to assess acceptability of the gel among HIV-positive men and their partners, the present study was limited in that the most comprehensive acceptability assessment among men was based on penile application of the gel. Men's responses may therefore not necessarily reflect acceptability during sexual intercourse. For similar reasons, the sexually abstinent women's acceptability responses may not be optimal. Furthermore, findings of this study cannot be generalized to all HIV-infected populations due to the unique characteristics, for example clinical status and sexual activity, of this population. Thus, while this study provides a snapshot of acceptability among HIV-positive men and women in Durban, a much larger study would be required to generalize acceptability outcomes to all HIV-positive individuals.

## Conclusion

This study of acceptability of a product that just completed large scale Phase III testing in South Africa provides valuable insight into desirable product characteristics among a select population group where these trials are conducted. Although these large studies will provide some information on HIV-negative women's perceptions of product characteristics, the present study advises on acceptability among women and men who are already HIV positive. This is critical as once an effective product is introduced onto the market, not all potential users are likely to know their HIV status, and therefore understanding acceptability of such products in all population groups is important. In general, Carraguard was found to be acceptable among the HIV-positive women and men in this study, with very few participants reporting side effects after use and most expressing generally favorable opinions about the product. However, certain product features, most notably gel volume, may require re-formulation (e.g. higher viscosity) to optimize product acceptability in certain populations or settings.

## List of Abbreviations

HIV Human Immunodeficiency Virus

AIDS Acquired Immune Deficiency Syndrome

IDI In-depth interview

STI Sexually transmitted infections

## Competing interests

The author(s) declare that they have no competing interests.

## Authors' contributions

GR was the principle investigator at the MRC for the study and worked with the Population Council to develop the protocol and systems for study implementation. She wrote the paper following data analysis.

NSM was the project leader of the study and wrote the paper with Prof. Ramjee and Ms. Braunstein. She analyzed the data and was responsible for data quality. She also implemented the study and developed SOPs with the team.

SB was the coordinator of this study at the Population Council. She helped develop study instruments, monitor study implementation, analyze the data, and wrote this paper with Prof. Ramjee and Ms. Morar.

BF worked on the original draft of the protocol, informed consent forms and study instruments as the first coordinator of this study at the Population Council. She advised on the qualitative data analysis for the study, and contributed editorial comments on several versions of this paper.

HJ was the data manager for this study at the Population Council, and worked on data analysis. She contributed editorial comments on several versions of this paper.

JvdW was the principle investigator of this study at the Population Council. She developed the original protocol and study instruments; oversaw study implementation at the Population Council; and contributed editorial comments on several versions of this paper.

All authors have read and approved this final manuscript.
